# Diabetic Cheiroarthropathy: A Case Report and Review of the Literature

**DOI:** 10.1155/2013/257028

**Published:** 2013-05-15

**Authors:** Rabia Cherqaoui, Sheldon McKenzie, Gail Nunlee-Bland

**Affiliations:** ^1^Division of Endocrinology and Metabolism, Howard University Hospital, 2041 Georgia Avenue NW, Washington, DC 20060, USA; ^2^Department of Internal Medicine, Howard University Hospital, Washington, DC 20060, USA

## Abstract

Diabetes mellitus is associated with a wide variety of rheumatologic manifestations which can significantly affect a patient's quality of life. One of these manifestations includes diabetic cheiroarthropathy (DCA) which affects the hands. We review a case of a 28-year-old female patient with type 1 diabetes mellitus who was diagnosed with DCA after complaining of limited movements of all joints in her hands and tightening of the skin. We examine how the diagnosis was made, the treatment administered, and the successful clinical outcome. Clinicians should be able to identify and treat this affliction. The diagnosis is mainly clinical. It is imperative to remember that the presence of DCA carries with it a significant relationship with microvascular disease.

## 1. Introduction

The syndrome of limited joint mobility, diabetic sclerosis, pseudosclerodermatous hand of the diabetic, and diabetic stiff hand are some of the diagnostic terms used in the medical literature which refer to diabetic cheiroarthropathy (DCA). It is usually characterized by painless limited extension of the proximal metacarpophalangeal joints and/or interphalangeal joints with spontaneous flexion of the fingers. There is decreased ability to fully flex or fully extend the fingers [1, 2]. The affliction can be associated with significant pain and stiffness. A tight waxy skin surface over the dorsum of the hand usually completes the clinical picture [3].

Diabetic cheiroarthropathy occurs in both type 1 and type 2 diabetes mellitus. Some DCA reports suggest an overall prevalence being quoted as 30% [1] while other studies give prevalence ranges from 8% to 50% [4, 5].

## 2. Case Presentation

A 28-year-old female with a 12-year history of type 1 diabetes mellitus reported pain and stiffness in both hands of one-year duration. The pain was described as dull and aching lasting throughout the day and worsening at night. She reported morning stiffness in her hands lasting for 5 minutes. She admitted limited movements of all joints in her hands with associated tightening of the skin. She denied any changes in the color of her skin in her fingers with cold weather. There waere no dysphagia, no dry eyes, and no dry mouth.

Of note, her glycemic control was poor with glycosylated hemoglobin (HbA1c) ranging between 8.5 and 10%. She was on an insulin pump. She was found to have nonproliferative diabetic retinopathy and microalbuminuria. She did not have any neuropathy. There was no family history of rheumatologic disease.

**Figure 1:**
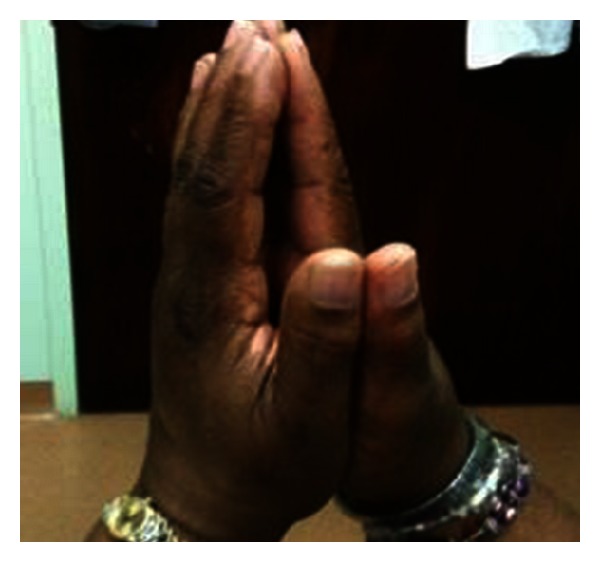
Positive prayer sign.

Diabetic cheiroarthropathy was diagnosed clinically after eliciting the “prayer” and “table top” signs. The prayer sign is said to occur whenever there is incomplete approximation of one or more of the digits when the patient attempts to approximate the palmar surfaces of the proximal and distal interphalangeal joints with palms pressed together and the fingers abducted (Figure 1). She was not able to completely lay her palms flat on a horizontal surface which denotes a positive tabletop sign.

There was no evidence of Duputren's contracture. Carpal tunnel syndrome was ruled out with negative Tinel's and Phalen's tests. There was no flexor tenosynovitis as evidenced by the absence of palpable crepitus.

Laboratory investigations such as erythrocyte sedimentation rate, C-reactive protein, rheumatoid factor, and other collagen vascular workup were normal. Radiographs of both hands showed mild prominence of proximal to mid interphalangeal joint soft tissue bilaterally.

The patient was placed on continuous glucose monitoring to improve her glycemic control. She was referred for physical and occupational therapy. Her symptoms improved within 6 months, although they did not completely resolve. She did report significant functional improvement.

## 3. Discussion

Diabetic cheiroarthropathy is a recognized complication of diabetes mellitus. It was initially described in patients with type 1 diabetic patients [6, 7] but has now been shown to occur in patients with type 2 diabetes mellitus as well. Some authors suggest that it occurs more frequently in type 1 diabetics because of the longer duration of their diabetes [8].

The genesis of the DCA is likely multifactorial with an interplay of a variety of factors. One possible mechanism is that the hyperglycemia facilitates the glycosylation and the crosslinking of collagen. Hence, the collagen proliferates extensively in the skin, subcutaneous tissues, tendons, muscles, and periarticular tissue. These collagen fibers become stiffer. Furthermore, there is decreased collagen degradation. Aside from the changes in collagen biochemistry, there is ongoing diabetic microangiopathy of the skin and subcutaneous vessels. There is also thickening of capillary basement membranes. As a consequence, there is low-grade ischemia of the tissues resulting in fibrosis manifesting as tight waxy skin over the digits. The epidermis and dermis are microscopically deficient in normal skin architecture because of the ischemia [9].

It was Rosenbloom and associates in 1981 who demonstrated a strong association between the increasing severity of joint limitation and the increased prevalence of microvascular disease in type 1 diabetes mellitus [7]. They reported a 3 fold risk of clinically apparent microvascular disease in patients with DCA. They concluded that limited joint mobility identifies a population that is, at increased risk for early-onset microvasculopathy. Subsequent studies have supported this finding [10, 11]. Our patient had evidence of nonproliferative retinopathy in addition to nephropathy at the time when she presented with symptoms related to her hands.

Is there a role for imaging in the diagnosis of DCA? The diagnosis is primarily a clinical one confirmed by the presence of the prayer and the table top signs. However, Ismail et al. concluded that ultrasonography easily demonstrates flexor sheath thickening in patients with DCA [12]. Ultrasound was also found to be reliable in excluding those who did not have the affliction based on clinical criteria. Serban and Udrea (2012) commented on the usefulness of Magnetic Resonance Imaging in detecting thickening and enhancement of flexor tendon sheaths [13].

With improved glycemic control, the symptoms and signs can be ameliorated. Some authors even suggest that complete reversal of DCA is possible. There have been reports in the literature to support this [14, 15]. Treatment relies primarily on glycemic control, in addition, to non-steroidal anti-inflammatory drugs and physical therapy. The literature does not define these treatments as specific to DCA [16]. If instituted early, treatment can improve patients' symptoms or reverse the clinical picture all together. Early consideration of the diagnosis can allow the clinician to make the link with microvascular complications and intervene accordingly. Our patient was placed on continuous glucose monitoring and subsequently her glycemic control improved. She also underwent physical and occupational therapy. Her hand symptoms improved dramatically.

## 4. Conclusion

It is important that clinicians be aware of diabetic cheiroarthropathy and its close relationship with metabolic control of diabetes and microvasculopathy. Patients with diabetes mellitus can be quickly screened for the classic signs of DCA. The condition is reversible in some patients.
